# The effect of a dedicated intensivist staffing to a medical emergency team on airway management in general wards

**DOI:** 10.1097/MD.0000000000038571

**Published:** 2024-06-21

**Authors:** Yehyeon Yi, Da-Hye Kim, Eun-Joo Choi, Sang-Bum Hong, Dong Kyu Oh

**Affiliations:** a Department of Pulmonology, Seoul Medical Center, Seoul, Republic of Korea; b Medical Emergency Team, Asan Medical Center, Seoul, Republic of Korea; c Department of Pulmonary and Critical Care Medicine, Asan Medical Center, University of Ulsan College of Medicine, Seoul, Republic of Korea; d Department of Pulmonology, Dongkang General Hospital, Ulsan, Republic of Korea.

**Keywords:** airway management, hospital rapid response team, intubation, rapid sequence induction and intubation

## Abstract

Although medical emergency teams (METs) have been widely introduced, studies on the importance of a dedicated intensivist staffing to METs are lacking. A single-center retrospective before-and-after study was performed. Deteriorating patients who required emergency airway management in general wards by MET were included in this study. We divided the study period according to the presence of a dedicated intensivist staff in MET: (1) non-staffed period (from January 2016 to February 2018, n = 971) and (2) staffed period (from March 2018 to December 2019, n = 651), and compared emergency airway management-related variables and outcomes between the periods. Among 1622 patients included, mean age was 63.0 years and male patients were 64.2% (n = 1042). The first-pass success rate was significantly increased in the staffed period (85.9% in the non-staffed vs 89.2% in the staffed; *P* = .047). Compliance to rapid sequence intubation was increased (9.4% vs 34.4%; *P* < .001) and vocal cords were more clearly open (*P* < .001) in the staffed period. The SpO_2_/FiO_2_ ratio (median [interquartile range], 125 [113–218] vs 136 [116–234]; *P* = .007) and the ROX index (4.6 [3.4–7.6] vs 5.1 [3.6–8.5]; *P* = .013) at the time of intubation was higher in the staffed period, suggesting the decision on intubation was made earlier. The post-intubation hypoxemia was less commonly occurred in the staffed period (7.2% vs 4.2%, *P* = .018). In multivariate analysis, the rank of operator was a strong predictor of the first-pass success (adjusted OR [95% CI], 2.280 [1.639–3.172]; *P* < .001 for fellow and 5.066 [1.740–14.747]; *P* < .001 for staff, relative to resident). In our hospital, a dedicated intensivist staffing to MET was associated with improved emergency airway management in general wards. Staffing an intensivist to MET needs to be encouraged to improve the performance of MET and the patient safety.

## 1. Introduction

The medical emergency teams (METs), also known as the hospital rapid response teams, have been widely introduced to intervene in the care of hospitalized patients with unexpected clinical deterioration.^[1,2]^ They monitor hospitalized patients and, if necessary, intervene in early stage of clinical deterioration and contribute to improving patient safety. The interventions performed by METs range from simple treatments to critical care interventions and end-of-life care planning which require highly trained and experienced medical personnels. However, in many hospitals, the METs are being operated without a dedicated or an attending intensivist staff^[3,4]^ and studies on the importance of an intensivist staffing to METs are lacking.

The emergency airway management is one of the major critical care interventions performed by METs.^[1,2,4,5]^ Its importance cannot be overemphasized because it is closely related to serious consequences and high morbidity and mortality in failed cases.^[6,7]^ Therefore, many efforts have been made to improve the quality of emergency airway management. For example, there have been reports that the airway management can be improved through appropriate training programs by experienced professionals.^[8–10]^ Recognizing difficult airway prior to intubation is also important in improving the quality of emergency airway management.^[7]^ In addition, the experience of operator is a well-known predicting factor of successful airway management.^[11,12]^ These studies show that experienced professionals are necessary to improve the quality of emergency airway management. However, there is a lack of data whether a dedicated intensivist staffing to MET improves the quality of emergency airway management.

Recently, in our hospital, we had an opportunity to staff a dedicated intensivist to MET. Therefore, by performing a before-and-after study, we aimed to investigate the effect of a dedicated intensivist staffing to MET on the clinical outcomes of emergency airway management in general wards.

## 2. Methods

### 2.1. Study design and data collection

A single-center retrospective before-and-after study was conducted at a tertiary hospital in Seoul, Korea. The hospital has 2700 beds and approximately 100,000 patients are admitted annually.^[13]^ The MET was introduced to the hospital in 2008 and has been operated 24 hours a day and 7 days a week since 2009. Until February 2018, the MET consisted of internal medicine residents, a critical care fellow and nurses and the airway management performed by residents was supervised by a critical care fellow. From March 2018, on the other hand, a dedicated intensivist staff has joined the team, and it began to be operated by internal medicine residents, a critical care fellow, an intensivist staff and nurses. In this period, the airway management performed by residents or fellows was supervised by an intensivist staff.

**Figure 1.**
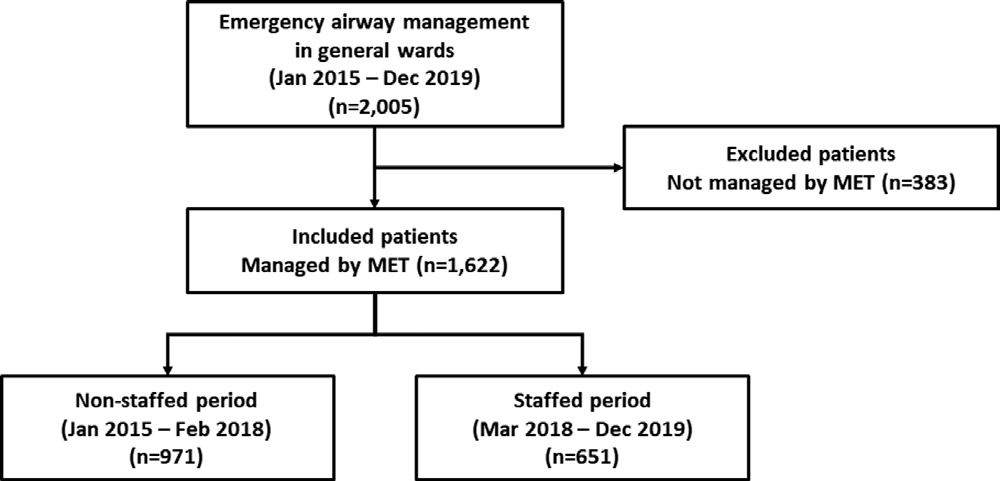
A flow diagram for patient inclusion and exclusion. MET = medical emergency team.

We included deteriorating adult patients (age ≥ 19 years) who required emergency airway management by MET in general wards between January 2015 and December 2019. The data were censored in December 2019, because thereafter the MET system has changed frequently because of the COVID-19 pandemic. We divided the study period according to the presence of a dedicated intensivist staff in MET: (1) non-staffed period (from January 2016 to February 2018, n = 971) and (2) staffed period (from March 2018 to December 2019, n = 651), and compared emergency airway management-related variables and outcomes between the periods (Fig. 1). Factors associated with the first-pass success were investigated as well.

Data on baseline characteristics were retrieved at the time of intubation, which include age, sex, presence of difficult airway, vital signs, mental status assessed by Glasgow coma scale, oxygenation-related variables (pulse oximeter oxygen saturation [SpO_2_], fraction of inspired oxygen [FiO_2_], SpO_2_/FiO_2_ ratio, and the ratio of pulse oximeter oxygen saturation (SpO_2_)/fraction of inspired oxygen (FiO_2_) to respiratory rate [ROX index]), and indications for intubation. The ROX index was calculated as SpO_2_/FiO_2_/respiratory rate.^[14]^ A lower ROX index means “worse” as it suggests a lower oxygen saturation, a higher FiO_2_ requirement, and tachypnea indicating impending respiratory failure requiring intubation. The quality of intubating condition was assessed by 2 of the 3 items of Cooper scoring system—jaw relaxation and vocal cords position—but response to intubation was not assessed in this study.^[15]^ Jaw relaxation was graded as follows: 0—impossible, 1—opens, 2—moderate, and 3—easy and vocal cords position was ranked as follows: 0—closed, 1—closing, 2—moving, and 3—open for vocal cords position.^[15]^ Data on emergency airway management-related variables and outcomes included rank of operator (resident, fellow, or staff), devices used for intubation (conventional laryngoscope or video-laryngoscope), compliance to rapid sequence intubation (RSI) protocol,^[16]^ jaw relaxation,^[15,17]^ vocal cords position,^[15,18]^ first-pass success,^[19]^ failed airway,^[16]^ time required for intubation, and post-intubation complications such as hypotension (systolic blood pressure < 90 mm Hg), hypoxemia (SpO_2_ < 90%), cardiac arrest, oral bleeding, and dental injury. The first-pass success was defined as a successful intubation after the first insertion of the laryngoscope blade into the mouth.^[19]^ The failed airway was defined as requiring 4 or more attempts at intubation or failure to insert the endotracheal tube.^[16]^

The study protocol was approved by the institutional review board of our hospital with the approval number of 2020-0286. The requirement for informed consent was waived because of the retrospective nature of the study and the use of anonymized clinical data.

### 2.2. Statistical analysis

Continuous variables were presented as mean ± standard deviation or median with interquartile range and compared using Student *t* test or Wilcoxon rank-sum test, as appropriate. Categorical variables were presented as number (%) and compared using chi-square or Fisher exact test, as appropriate. A logistic regression model was used to assess the factors associated with first-pass success and the association was presented as odds ratio and 95% confidence interval. Univariate analyses were initially performed to identify potentially associating factors and those with *P* < .10 were included in the multivariate analysis. The multicollinearity effects of associating factors were assessed using variance inflation factors with a cutoff value of >10. All statistical analyses were two-sided, and *P* < .05 was considered significant. Statistical analyses were performed using the Statistical Package for Social Science (SPSS) version 22.0 for Windows (IBM Corporation, Armonk, NY).

## 3. Results

### 3.1. Comparisons of baseline characteristics between the non-staffed and the staffed periods

**Figure 2.**
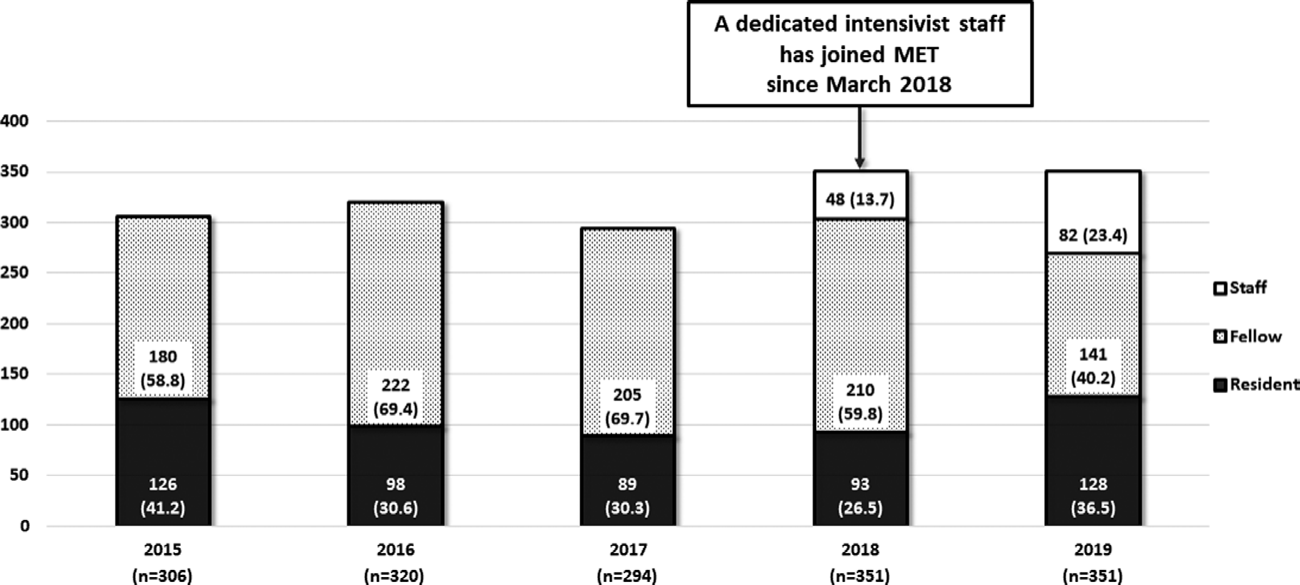
Annually performed emergency airway management by MET in general wards. Note that a dedicated intensivist staff joined MET in March 2018 and the proportion of patients directly managed by the intensivist staff increased up to 23.4% in 2019. MET = medical emergency team.

During the study period, a total of 2005 adult patients received emergency airway management in general wards. Of these, 383 cases were excluded because they were not managed by MET. A total of 1622 cases were included in the study (Fig. 1) and as shown in Figure 2 each year approximately between 300 and 350 adult patients received emergency airway management by MET in general wards. The proportion of patients directly managed by a dedicated attending intensivist staff was 13.7% in 2018 and increased to 23.4% in 2019.

In the non-staffed and the staffed periods, 971 and 651 adult patients received emergency airway management in general wards, respectively (Fig 1 and Table 1). The mean age of included patients was 63.0 years and male patients were 64.2% (n = 1042). There were no differences in terms of demographics between the periods. Difficult airway was assessed in 18.9% (n = 306) and no difference was observed between the periods. Interestingly, however, the oxygenation-related variables at the time of intubation were significantly different between the periods. The proportion of patients with SpO_2_ < 90% was lower in the staffed period (24.1% in the non-staffed period vs 18.0% in the staffed period; *P* = .007). In addition, both the median SpO_2_/FiO_2_ ratio (125 [113–218] vs 136 [116–234]; *P* = .007) and the median ROX index (4.6 [3.4–7.6] vs 5.1 [3.6–8.5]) were higher in the staffed period. The mean Glasgow coma scale was higher in the staffed period as well (9.9 ± 5.2 vs 11.0 ± 4.8; *P* < .001).

**Table 1:** Comparisons of baseline characteristics between the non-staffed and the staffed periods.

Variables	Non-staffed period (n = 971)	Staffed period (n = 651)	*P*-value
Age, years	62.5 ± 14.4	63.6 ± 13.8	.118
Male	622 (64.1)	420 (64.5)	.850
Difficult airway	177 (18.2)	129 (19.8)	.423
Vital signs			
Systolic blood pressure, mm Hg	123.4 ± 33.5	126.4 ± 33.2	.112
Diastolic blood pressure, mm Hg	71.1 ± 21.2	72.3 ± 20.7	.279
Heart rates/min	118.3 ± 26.4	118.4 ± 26.5	.941
Respiratory rates/min	31.1 ± 8.7	30.6 ± 8.5	.563
Oxygenation-related variables			
SpO_2_, %	95.0 (90.0–98.0)	96.0 (91.0–99.0)	<.001
SpO_2_ < 90%	188 (24.1)	99 (18.0)	.007
FiO_2_, %	61.0 (35.0–80.0)	60.0 (29.0–80.0)	.249
SpO_2_/ FiO_2_ ratio	125 (113–218)	136 (116–234)	.007
ROX index*	4.6 (3.4–7.6)	5.1 (3.6–8.5)	.013
Glasgow coma scale	9.9 ± 5.2	11.0 ± 4.8	<.001
Indications for intubation			
Hypoxemia	454 (46.8)	279 (42.9)	.122
Hypercapnia	76 (7.8)	68 (10.4)	.069
Cardiac arrest	171 (17.6)	93 (14.3)	.075
Airway protection	116 (11.9)	72 (11.1)	.585
Increased work of breathing	144 (14.8)	132 (20.3)	.004
Others	10 (1.0)	7 (1.1)	.930

Data were presented as number (%), mean ± standard deviation, or median (interquartile range) as appropriate.

FiO_2_ = fraction of inspired oxygen, SpO_2_ = pulse oximeter oxygen saturation.

* ROX index = SpO_2_/FiO_2_/respiratory rates.

### 3.2. Comparisons of emergency airway management-related variables and outcomes between the non-staffed and the staffed periods

Table 2 shows the differences in the emergency airway management between the periods. In the staffed period, the operator used video-laryngoscope more frequently during intubation (88.2% vs 97.1%; *P* < .001). The compliance to RSI protocol was significantly increased in the staffed period (9.4% vs 34.4%; *P* < .001) and, interestingly, these changes were observed in all ranks of operators (9.4% vs 34.4%; *P* < .001 in residents and 8.2% vs 15.7%; *P* < .001 in fellows). In addition, as compliance to the RSI protocol improved, the jaw was more easily relaxed (*P* < .001) and the vocal cords were more clearly open (*P* < .001) during intubation in the staffed period (Table 2).

**Table 2:** Comparisons of emergency airway management-related variables and outcomes between the non-staffed and the staffed periods.

Variables	Non-staffed period (n = 971)	Staffed period (n = 651)	*P*-value
Use of video-laryngoscope	854 (88.2)	631 (97.1)	<.001
Compliance to RSI protocol	91 (9.4)	224 (34.4)	<.001
Resident	39/337 (11.6)	94/197 (47.7)	<.001
Fellow	52/634 (8.2)	51/324 (15.7)	<.001
Staff	NA	79/130 (60.8)	NA
Jaw relaxation			<.001
Easy	803 (83.0)	626 (96.5)	
Moderate	140 (14.5)	19 (2.9)	
Opens	25 (2.6)	4 (0.6)	
Impossible	0 (0.0)	0 (0.0)	
Vocal cords position			<.001
Open	873 (90.3)	617 (95.1)	
Moving	75 (7.8)	25 (3.9)	
Closing	16 (1.7)	4 (0.6)	
Closed	3 (0.3)	3 (0.5)	
First-pass success	834 (85.9)	581 (89.2)	.047
Resident	269/337 (79.8)	169/197 (85.8)	.083
Fellow	565/634 (89.1)	286/324 (88.3)	.694
Staff	NA	126/130 (96.9)	NA
Failed airway	15 (1.5)	12 (1.8)	.645
Time to intubation, minutes	3.0 (2.0–5.0)	3.0 (2.0–4.0)	.362
Time to intubation > 5 minutes	141 (14.5)	68 (10.8)	.028
Post-intubation complications			
Hypotension*	156 (16.1)	197 (14.9)	.526
Hypoxemia†	61 (7.2)	24 (4.2)	.018
Cardiac arrest	3 (0.3)	1 (0.2)	.653
Others‡	15 (1.5)	13 (2.0)	.493

Data were presented as number (%) or median (interquartile range) as appropriate.

NA = not available, RSI protocol = rapid sequence intubation protocol, SpO_2_ = pulse oximeter oxygen saturation.

* Hypotension was assessed as systolic blood pressure less than 90 mmHg.

† Hypoxemia was assessed as SpO_2_ less than 90%.

‡ Others include oral bleeding and dental injury.

For the outcome variables, the first-pass success rate was increased in the staffed period (85.9% vs 89.2%; *P* = .047). In the resident group, the first-pass success rate tended to improve in the staffed period (79.8% vs 85.8%; *P* = .083), however there was no significant difference in the fellow group (89.1% vs 88.3%; *P* = .694). The median time required for intubation was 3 minutes and there was no difference between the periods. However, the proportion of patients who required > 5 minutes for intubation was significantly lower in the staffed period (14.5% vs 10.8%; *P* = .028). Furthermore, post-intubation hypoxemia defined as SpO_2_ < 90% was less common in the staffed period (7.2% vs 4.2%; *P* = .018).

### 3.3. Factors associated with first-pass success

Table 3 shows the factors associated with first-pass success. In the univariate analyses, the presence of difficult airway (inversely related), rank of operator, use of video-laryngoscope, compliance to RSI protocol, and the staffed period were significantly associated with first-pass success. In the multivariate analysis, the rank of operator was a strong predictor of the first-pass success (adjusted OR [95% CI], 2.280 [1.639–3.172]; *P* < .001 for fellow and 5.066 [1.740–14.747]; *P* < .001 for staff, relative to resident), indicating that when the intensivist staff performs intubation the possibility of first-pass success increases by >5 times compared to when the resident performs. The use of video-laryngoscope (6.904 [4.169–9.010]; *P* < .001) and compliance to RSI protocol (3.876 [2.155–6.969]; *P* < .001) were also associated with the first-pass success (Table 3) and both were significantly increased in the staffed period (Table 2).

**Table 3:** Factors associated with first-pass success.

Univariate analysis	Unadjusted OR	95% CI	*P*-value
Age	1.006	0.996–1.016	.267
Male (vs female)	0.841	0.617–1.148	.276
Difficult airway	0.335	0.244–0.459	<.001
Rank of operator			
Resident	1.000	NA	Reference
Fellow	1.743	1.293–2.350	<.001
Staff	6.904	2.491–19.139	<.001
Use of video-laryngoscope (vs direct laryngoscope)	6.129	4.169–9.010	<.001
Compliance to RSI protocol (vs noncompliance)	3.444	2.006–5.914	<.001
Staffed period (vs non-staffed period)	1.363	1.003–1.853	.048
**Multivariate analysis**	**Adjusted OR** *	**95% CI**	***P*-value**
Difficult airway	0.263	0.185–0.373	<.001
Rank of operator			
Resident	1.000	NA	Reference
Fellow	2.280	1.639–3.172	<.001
Staff	5.066	1.740–14.747	<.001
Use of video-laryngoscope (vs direct laryngoscope)	6.932	4.541–10.583	<.001
Compliance to RSI protocol (vs noncompliance)	3.876	2.155–6.969	<.001
Staffed period (vs non-staffed period)	0.722	0.507–1.028	.071

CI = confidence interval, NA = not available, OR = odds ratio, RSI protocol = rapid sequence intubation protocol.

* Adjusted for difficult airway, rank of operator, use of video-laryngoscope, compliance to RSI, and staffed period.

## 4. Discussion

In this retrospective before-and-after study, we found that a dedicated intensivist staffing may have a positive effect on the emergency airway management performed by MET in general wards. First of all, the first-pass success which is an important hard-outcome in many airway management studies,^[19,20]^ was significantly increased after a dedicated intensivist staffing. Moreover, in the staffed period, the decision on intubation was made earlier, the intubation was more frequently performed by experienced operators, the video-laryngoscope and the RSI protocol were used more frequently, and the post-intubation complications were less commonly occurred. These suggest that the overall quality of emergency airway management has improved after a dedicated intensivist staffing to MET and indicate that it may contribute to the improved patient safety in general wards.

Although the METs have been widely introduced, the optimal composition of team is still uncertain and whether the presence of dedicated doctors within the METs leads to better outcomes remains controversial.^[4]^ In a previous multi-center retrospective observational study, the presence of dedicated doctors within the METs was not associated with better survival in the overall population, but with better survival and lower intensive care unit admission rates among patients with sepsis or septic shock.^[4]^ However, the study did not address the outcomes related to emergency airway management. To the best of our knowledge, this study is the first to investigate the association between a dedicated intensivist staffing to MET and the emergency airway management and to show that it is associated with improved quality of emergency airway management and the patient safety. Considering that there is still controversy over the composition of MET, our findings can provide a reference for the optimal composition of the team. And it also provides a reference for the MET-related health care policies such as healthcare insurance policy.

Since the increased number of intubation attempts is associated with adverse events and poor prognosis, the first-pass success rate is widely accepted as a reasonable indicator for the quality of emergency airway management.^[19,20]^ In our study, the first-pass success rate was 85.9% in the non-staffed period and was significantly increased to 89.2% in the staffed period (*P* = .047). The resident group showed a tendency to improve the first-pass success rate (79.8% vs 85.8%; *P* = .083), however the fellow group did not show significant difference (89.1% vs 88.3%; *P* = .694) (Table 2). Considering that the first-pass success rates in the previous studies which include deteriorating patients in general wards were approximately 75%,^[21,22]^ it seems that the marginal improvement may stem from the relatively higher first-pass success rate in the non-staffed period than in the previous studies. And we believe that these findings indicate even in such a well-established MET, a dedicated intensivist staffing may archive a positive effect on improving the quality of airway management.

It is well-known that the delayed intubation is associated with higher incidence of post-intubation complication and poor prognosis such as higher intensive care unit and hospital mortalities.^[14,23,24]^ Therefore, for MET physician, it is critical to decide when to perform the intubation. Recent studies have suggested that the ROX index of 4.88 may be a reference value when determining intubation in patients with high flow nasal cannula.^[14,24]^ In this study, the median ROX index at the time of intubation in the staffed period was increased to 5.1 (Table 1), which are comparable to the previously suggested reference value of 4.88. Furthermore, the incidence of post-intubation hypoxemia was decreased in the staffed period (Table 2). Although this study does not include the data on mid- to long-term outcomes such as intensive care unit and hospital mortalities, it showed that a dedicated intensivist staffing to MET may be associated with the timely intubation and the decreased immediate post-intubation complication such as hypoxemia.

In the recent studies, the use of video-laryngoscope has been considered a key factor in improving the quality of emergency airway management^[25]^ and is recommended by the guidelines.^[26]^ In addition, it is generally recommended to use the RSI protocol during intubation, which includes the administration of an induction agent followed by a neuromuscular blocking agent, to create the optimal intubating conditions and minimized the airway unprotection time.^[27–29]^ In our study, the use of video-laryngoscope and the compliance to RSI protocol were significantly increased in the staffed period (Table 2) and both were independently associated with the first-pass success (Table 3). Moreover, the compliance to RSI protocol was increased in both resident and fellow groups in staffed period (Table 2), suggesting the dedicating intensivist staffing to MET is associated with the improved resident and fellow training program as well.

In the previous studies, the experience of operator was an independent predictor of the first-pass success in emergency room or in operating room.^[11,30–32]^ However, the data from deteriorating patients in general wards were lacking. In our study, we showed that the rank of operator is a significant predictor of the first-pass success and, particularly, when the intubation was performed by an intensivist staff, the possibility of first-pass success was >5 times higher than that was performed by an internal medicine resident (Table 3). These findings are in line with the previous studies performed in other suites, and suggest that the METs require dedicated intensivist staffs, as there are emergency medicine staffs in the emergency department and anesthesiologist staffs in the operating rooms.

This study has several limitations. First, since the study was conducted with retrospective nature, there were missing values such as response to intubation in Cooper scoring system and there may be inherent risks of bias those were not fully addressed. In addition, because of the before-and-after study design which compares the airway management related outcomes between the non-staffed and the staffed periods, the influence of various confounding factors may not have been fully adjusted. Secondary, because the study was conducted in a single center, it is unclear whether the findings in our study would be observed in other hospitals. Moreover, considering that our hospital already had well-established MET even in the non-staffed period, we believe that the findings in our study should be interpreted with caution and the following prospective multi-center studies are necessary to confirm these findings. Lastly, since the study does not include the data on mid- to long-term outcomes such as mortality and ventilator weaning, we could not assess the relationship between the intensivist staffing to MET and these outcomes. Following studies are required to address these issues.

In conclusion, in our hospital, a dedicated intensivist staffing to MET was significantly associated with improved the quality of emergency airway management in general wards. Staffing a dedicated intensivist to MET needs to be encouraged to improve the performance of MET and the patient safety.

## Author contributions

**Conceptualization:** Sang-Bum Hong, Dong Kyu Oh.

**Data curation:** Yehyeon Yi, Da-Hye Kim, Eun-Joo Choi, Sang-Bum Hong, Dong Kyu Oh.

**Formal analysis:** Yehyeon Yi, Dong Kyu Oh.

**Methodology:** Yehyeon Yi, Da-Hye Kim, Eun-Joo Choi, Dong Kyu Oh.

**Visualization:** Yehyeon Yi, Da-Hye Kim, Dong Kyu Oh.

**Writing – original draft:** Yehyeon Yi, Dong Kyu Oh.

**Writing – review & editing:** Yehyeon Yi, Da-Hye Kim, Eun-Joo Choi, Sang-Bum Hong, Dong Kyu Oh.
